# Survival rate and predictors of outcome in intubated patients with haematological malignancies in a Greek ICU

**DOI:** 10.1186/cc13230

**Published:** 2014-03-17

**Authors:** V Tsolaki, M Karapetsa, G Ganeli, A Mpouzia, E Zakynthinos

**Affiliations:** 1 University Hospital of Larissa, Greece

## Introduction

The admission of patients with haematological malignancies to the ICU is associated with high mortality, especially when mechanical ventilation is required [1,2]. The purpose of the present study was to explore survival rates and the predictors of survival in this group of patients.

## Methods

A retrospective study of intubated patients with haemato- logical malignancies, admitted to a general ICU in central Greece, due to any cause.

## Results

During a 10-year period (2003 to 2013), 16 patients with haematological malignancies (nine with acute myelogenous leukaemia, four with non-Hodgkin lymphoma, one with Hodgkin lymphoma and two with multiple myeloma, mean age 50.75 ± 16.59) (male/ female 3/13, mean ApACHE II score 23.18 ± 6.67, mean SOFA score 12.50 ± 2.82, CRP 16.00 ± 10.13, WBC 2.055 ± 30.053) were admitted to the ICU. The majority of patients were admitted due to ARDS (PO_2_/ FiO_2 _164 ± 109), one patient was admitted due to intestinal rupture and peritonitis and the other one due to intracerebral haemorrhage. In the majority of the patients (13/16) diagnosis of the malignancy was made during the present admission and only three had the malignancy for a longer period (5 months to 3 years). The mean ICU length of stay was 10.56 ± 16.19 days. A total 68.75% (11/16) of the patients were intubated upon admission, whereas the mean time to intubation for the rest of the patients was 6.37 ± 4.16 hours. Neither intubation upon admission nor time to intubation was correlated with survival. Type of haematological malignancy, duration of immunosuppression and preceding length of stay in the general ward did not correlate with survival either. The survival rate was 18.7% and in linear regression analysis, duration of treatment with NIV in the general ward, increased SOFA score and the number of platelets (<50.000) upon admission to the ICU were independent predictors of survival (*R*^2 ^= 0.77, *P *= 0.017).

## Conclusion

The present retrospective study indicates that patients with haematological malignancies have poor survival when they are admitted to the ICU. Longstanding treatment with NIV before ICU admission, high SOFA scores and low platelet levels upon admission negatively affect survival.
